# *Scutellaria baicalensis and Lonicera japonica* herbal extracts enhanced the performance and physiological responses of pigs subjected to hot and dry season

**DOI:** 10.3389/fvets.2025.1740443

**Published:** 2026-03-24

**Authors:** Vetriselvi Sampath, Yu Jin Baek, In Ho Kim

**Affiliations:** Department of Animal Resources, Dankook University, Cheonan, Republic of Korea

**Keywords:** grow-finishing pigs, heat stress, nutrient digestibility, oxidative stress, rectal temperature, gut health

## Abstract

A 10-week growth trial was conducted using three groups of 70 growing-finishing pigs [210 head (Landrace × Yorkshire ♀ × Duroc ♂) with initial BW 53.92 ± 2.38 kg] to evaluate the effects of herbal extract mixture (HEM), composed of *Scutellaria baicalensis* and *Lonicera japonica* on overall performance of growing–finishing pigs. The pens of pigs (5 pigs/pen- 3 gilts and 2 barrows) were randomly allocated into one of three treatment groups in a completely randomized design with 14 replications per treatment based on their initial BW and sex. The dietary treatments were as follows: (1) basal diet (CON), (2) basal diet supplemented with 05, and 0.10% of HEM as TRT1 and 2. Diets were offered in two phases: Phase 1 (weeks 0–5) and Phase 2 (weeks 5–10). During Phase 1, pig fed increasing HEM supplement had tended to improve G: F compared to CON. Also, during phase 2 and overall experimental period pigs fed increasing HEM supplement had linearly increased BW, daily gain, and daily feed intake and tendency to improve gain to feed ratio. Also showed linearly improved nitrogen digestibility. At week 5, pigs receiving HEM had lower respiratory rates and rectal temperatures, indicating improved heat stress tolerance. Furthermore, HEM treated pigs showed linearly reduced cortisol levels and HSP70 level and tend to reduce MDA level suggesting lower stress and oxidative damage. Biochemical markers such as glucose, insulin, packed cell volume, superoxide dismutase, and glutathione peroxidase were elevated in pigs fed increasing HEM supplementation, while blood urea nitrogen and immune cells remained unaffected. Alpha and beta diversity were assessed and across all indices, and no significant differences were observed among treatment groups. However, the relative abundance of phyla *Firmicutes* and *Bacteroidota* were significantly higher and relative abundance of genus *Prevotella* were significantly lower in HEM treated pigs suggesting the herbal extract alleviate the intestinal inflammation in pigs during hot and dry seasons. In summary, adding 0.05 and 0.10% HEM to growing-finishing pig diet appears to be beneficial to support the overall health and enhance production during hot and dry climates.

## 1 Introduction

Heat stress (HS) has become a major challenge in modern swine production, especially in tropical and subtropical regions where elevated temperatures and humidity significantly compromise animal health, welfare, and productivity. With global temperatures continuing to rise, the frequency and severity of HS are expected to increase, posing substantial threats to the economic sustainability of livestock systems (1). Swine are particularly vulnerable due to their limited thermoregulatory capacity stemming from the lack of functional sweat glands and relatively low pulmonary efficiency which makes them highly susceptible to heat-induced physiological and metabolic disturbances (2). Consequently, HS in pigs often leads to reduced feed intake, impaired growth performance, increased morbidity and mortality, and considerable economic losses (3). To counteract these detrimental effects, conventional production systems have widely used synthetic growth promoters such as *β*-adrenergic agonists and antibiotics (4). However, mounting concerns regarding their public health, environmental impact, and contribution to antimicrobial resistance have led to strict regulations or bans in many countries (5). This shift has stimulated interest in alternative, sustainable strategies to improve animal performance under HS conditions. Among these, phytochemicals plant-derived bioactive compounds have gained considerable attention for their antioxidant, anti-inflammatory, and immune-supporting properties (6). *Scutellaria baicalensis* (Baikal skullcap) and *Lonicera japonica* (Japanese honeysuckle) herbs are rich in flavones, glycosides, and organic acids, and known for potent antioxidant and anti-inflammatory effects (7, 8). Several studies suggest that combining herbal extracts may enhance biological efficacy compared to using individual herbs alone (9, 10). For instance, Shang et al. (8) reported that dietary *Lonicera japonica* improved the immune response and feed intake of the host by clearing away their body heat. Also, Hosokawa et al. (11) reported the flavones, a primary component in these herbs, prevent the expression of heat shock proteins. Similarly, Wang et al. (12) reported that dietary supplementation with *L. flos* and *S. baicalensis* extracts at 0.5 g/kg during gestation has significantly improved reproductive performance and metabolic health in sows. Despite these promising observations, to date very limited study has evaluated the combined use of *S. baicalensis* and *L. japonica* in pigs, particularly during the hot and humid season. Therefore, the present study aims to address this gap by systematically evaluating the effects of an herbal extract mixture (HEM) containing *S. baicalensis* and *L. japonica* on physiological responses, health indicators, and production performance of growing–finishing pigs exposed to natural HS conditions.

## 2 Materials and methods

### 2.1 Ethical endorsement

All procedures involving animal handling were complied with ARRIVE guidelines and this research proposal was reviewed and approved (approval no: DK-1-2427) by Dankook University (DKU), Institutional Animal Care and Use Committee, prior to the trail.

### 2.2 Experimental facilities

**Figure 1:**
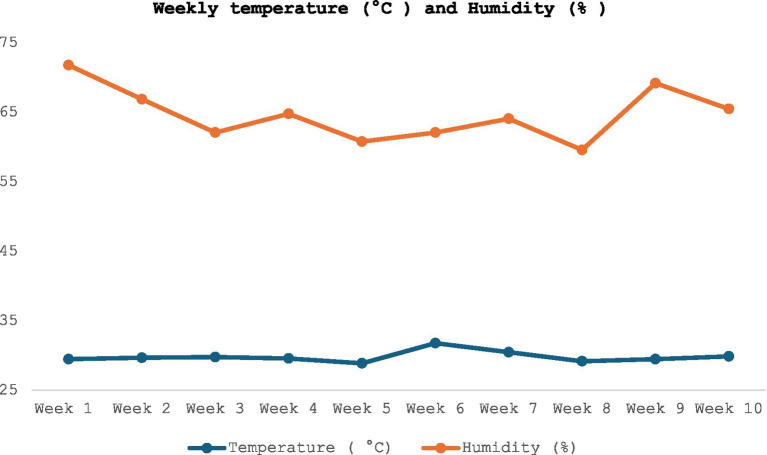
The ambient temperature and humidity of the experimental facility is shown in red and blue lines. Herein, the red line represents temperature (°C), while the blue line represents humidity (%).

The experiment was commenced during summer season at the “Experimental Facility” of DKU, located in Sejong-city, South Korea. The facility consisted of a double curtain-sided structure measuring 10 × 18 ft, equipped with pit fans for minimum ventilation and fully slated flooring (2.44 m × 1.53 m) positioned above a deep manure pit for waste management. Each pen (3.0 × 5.5 m, 0.6 m^2^/pig) was furnished with a nipple-type drinker and a single-hole self-feeder to allow pigs ad libitum access to water and experimental diets. The ambient temperature and relative humidity during this study was illustrated in Figure 1.

### 2.3 Experimental diets

The corn soybean based basal diet (mash form) was formulated to meet or exceed the National Research Council (13) recommendations for pig’s appropriate weight ranges (Table 1) With respect to HEM, (NO-STESOL®, Semi Feed Tech Co., Ltd. Korea the Republic), dried *S. baicalensis* and *L. japonica* herbs were procured from a local traditional Korean medicine market. Prior to extraction, plant materials (excluding *Lonicera japonica* seeds) were thoroughly washed, cleaned, and oven-dried using a conventional drying method. The dried herbs were then chopped and ground to pass through a 100-mesh sieve (approximately 2 mm). Extraction was performed using 200 L of 70% methanol at room temperature for 24 h in a large-scale extractor (Co-Biotechk, Seoul, Korea). The resulting methanolic extracts were filtered 2–3 times through cheesecloth to remove residues, followed by vacuum evaporation to concentrate the filtrates. These concentrates were subsequently freeze-dried and pulverized into fine powder extracts. The final product comprised of 55% *S. baicalensis,* 25% *L. japonica* extract, with 20% wheat bran served as a carrier. The bioactive components and proposed mechanisms of action for each herb are summarized in Table 2. The basal diet was prepared at the Laboratory of Swine Nutrition and Feed Technology (DKU) and subsequently divided into 3 bags with equal portions. The bag without any additive was labeled CON and the remaining two bags were labeled HEM 1 and 2.

**Table 1:** Ingredients and nutrient level of the complete diet of growing-finishing pigs (as fed basis %).

Ingredients (%)	Phase 1 (week 0–5)	Phase 2 (week 5–10)
Corn	77.81	83.46
Soybean meal	16.90	11.70
Tallow	0.44	0.17
Molasses	2.00	2.00
Monocaluium phosphate	0.90	0.80
Limestone	0.75	0.66
Lysine (78%)	0.35	0.36
Methionine (98%)	0.03	0.02
Threonine (98%)	0.11	0.11
Tryptophan (98%)	0.01	0.02
Salt	0.20	0.20
Mineral mix^1^	0.20	0.20
Vitamin mix^2^	0.20	0.20
Choline (25%)	0.10	0.10
Total	100.00	100.00
Analyzed nutrient level		
Crude protein, %	15.08	12.94
Calcium, %	0.47	0.64
Phosphorus, %	0.61	0.48
Lysine, %	1.10	0.87
Methionine, %	0.14	0.19
Threonine, %	0.75	0.49
Tryptophan, %	0.19	0.10
Crude Fat, %	3.28	3.11
Crude Fiber, %	2.18	2.25
Crude Ash, %	3.88	3.34

^1^Provided per kg diet: Fe, 100 mg as ferrous sulfate; Cu, 17 mg as copper sulfate; Mn, 17 mg as manganese oxide; I, 0.5 mg as potassium iodide; and Se, 0.3 mg as sodium selenite. ^2^Provided per kilograms of diet: vitamin A, 10,800 IU; vitamin D3, 4,000 IU; vitamin E, 40 IU; vitamin K3, 4 mg; vitamin B1, 6 mg; vitamin B2, 12 mg; vitamin B6, 6 mg; vitamin B12, 0.05 mg; biotin, 0.2 mg; folic acid, 2 mg; niacin, 50 mg; D-calcium pantothenate, 25 mg.

**Table 2:** Illustrates the main active components of *Scutellaria baicalensis* and *Lonicera japonica.*

Herbs	*Scutellaria baicalensis*	*Lonicera japonica*
Common name	Chinese skullcap 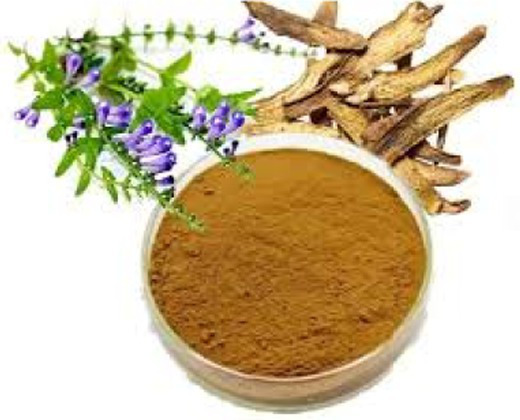	Japanese Honeysuckle 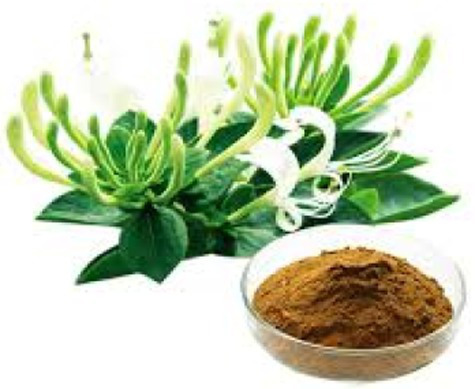
Family	Lamiaceae	Caprifoliaceae
Genus	Scutellaria	Lonicera
Active components and compounds	Flavones—Baicalin (10.30%), Baicalein (1.54%), oroxylin A	Organic acid (8.05 mg/g)- chlorogenic acid, isochlorogenic acid, caffeic acid flavones—chrysoeriol, luteolin iridoids (23%)—loganin saponins (80.01 mg/g)—loniceroside, hederagenin
Target effect	Reduce heat stress improve antioxidant anti-allergic, anti-tumor, anti-inflammatory effect (11, 57)	Reduce heat stress enhance the antioxidative, anti-tumor, anti-inflammatory, anti-bacterial, and anti-viral effect (11, 57).

### 2.4 Animals and study design

A 10-week growth trial was conducted using three groups of 70 growing-finishing pigs [210 head (Landrace × Yorkshire ♀ × Duroc ♂) with initial BW 53.92 ± 2.38 kg]. The pens of pigs (5 pigs/pen- 3 gilts and 2 barrows) were randomly allocated into one of three treatment groups in a completely randomized design with 14 replications per treatment based on their initial BW and sex. The dietary treatments were as follows: (1) basal diet (CON), (2) basal diet supplemented with 05, and 0.10% of HEM as TRT1 and 2. The test diets were fed in two phases: Phase 1, from week 0–5 and Phase 2, from week 5–10.

### 2.5 Sampling

The heaviest pigs in each pen were weighed individually, and feed intake was recorded on a pen basis at initial, phase 1, 2, and overall trial period to determine average daily gain (ADG, kg), daily feed intake (ADFI, kg), and gain-to-feed ratio (G: F). To assess nutrient digestibility, 0.3% chromium oxide was incorporated into the diets at the end of week 9 as an indigestible marker for the determination of apparent total tract digestibility (ATTD) of dry matter (DM), nitrogen (N), and energy (E). Representative feed samples were collected immediately following chromium oxide inclusion, sealed in zipper-lock bags, and transported to the laboratory for analysis. At the end of week 10, fresh fecal samples were obtained from two pigs (1 male and 1 female) per pen via rectal palpation. Samples were immediately placed on ice, transported to the laboratory, and stored at −15 °C for further analysis. Respiratory rate (RR) and Rectal Heat (RH) were measured (morning and evening) at the end of each phase. RR was determined by visually counting flank movements for a 60-s interval while the pigs were at rest, using a stopwatch, and recorded in breaths per minute (bpm) by a consistent observer. Blood samples (10 mL) were aseptically collected from the anterior vena cava of two pigs/pen (both sex) at the end of week 10 using sterile needles and syringes and the specimens were dispensed into two types of tubes: one containing ethylenediaminetetraacetic acid (EDTA) as an anticoagulant and the other without any anticoagulant. Tubes without EDTA were centrifuged at 10,000 *g* for 10 min to separate the serum, which was then stored at −20 °C until further analysis. Following the 10-week feeding trial, the same pigs were slaughtered after a 16-h fasting period to assess carcass characteristics and quality grades. All procedures were conducted in accordance with the guidelines and regulations established by the IACAUC.

### 2.6 Laboratory analyses

Crude protein (976.05), crude fat (942.05), crude fiber (973.18), and crude ash (942.05) in the basal diet were determined according to the association of official analytical collaboration. For nutrient digestibility analysis, fecal samples were oven-dried at 80 °C for 72 h, then ground to pass through a 1-mm sieve along with representative feed samples. Dry matter (DM) content was measured using Association of Official Analytical Chemists Method 930.5, while nitrogen (N) content was assessed using Method 978.04. Chromium concentration in both feed and feces was quantified using UV absorption spectrophotometry (Shimadzu UV-1201, Kyoto, Japan). The E was determined by bomb calorimetry (Parr 6,100; Parr Instrument Co., Moline, IL, United States) and protein (*N* × 6.25) content was determined by using a Kjeltec 2,300 Analyzer (Foss Tecator™, Hoeganaes, Sweden). The ATTD was calculated using the following equation: ATTD = [1 − (Nf × Cd)/(Nd × *Cf*)] (14). RH was recorded using a calibrated digital thermometer (GLA M700, GLA Agricultural Electronics, San Luis Obispo, CA, United States) with an accuracy ± 0.1 °C. Serum cortisol (Catalog No. MBS701325) and insulin (Catalog No. MBS4500450) levels were measured using enzyme-linked immunosorbent assay (ELSA) kits (Sigma-Aldrich^®^, St. Louis, MO, United States). The specimens stored in EDTA-treated tubes were used for the analysis of plasma glucose, blood urea nitrogen (BUN), white blood cell (WBC) count, and lymphocyte levels. Plasma glucose was quantified using a biochemical diagnostic analyzer (Vitros DTII), whereas BUN, WBC, and lymphocytes were analyzed with an automatic biochemical analyzer (Hitachi 7,600, Tokyo, Japan). Neutrophil (NE) levels were measured following the method described by Niekamp et al. (15) Whole blood samples were analyzed for packed cell volume (PCV) using a calibrated automated hematology analyzer (scil Vet abc, F-67120 Altorf, France). Serum levels of antioxidant enzymes including superoxide dismutase (SOD, Catalog No. A001-3), glutathione peroxidase (GPx, Catalog No. A005), and malondialdehyde (MDA, Catalog No. A003–1) were determined using commercial assay kits according to the manufacturer’s protocols (16). Serum concentrations of heat shock protein 70 (HSP70) were quantified using a microplate reader (Multiskan MK3, Waltham, MA, United States). Carcass grade and backfat thickness were evaluated based on the methodologies described by Muniyappan et al. (17).

Total genomic DNA was extracted from each sample using the QIAamp Fast DNA Stool Mini Kit (QIAGEN, Hilden, Germany) according to the manufacturer’s protocol and eluted in 100 μL of the kit-provided buffer. DNA concentration and purity were measured using a NanoDrop spectrophotometer. The V3–V4 region of the bacterial 16S rRNA gene was amplified using primers (forward: 5′-TCGTCGGCAGCGTCAGATGTGTATAAGAGACAG CCTACGGGNGGCWGCAG-3′; reverse: 5′-GTCTCGTGGGCTCGGAGATGTGTATAAGAGACAG GACTACHVGGGTATCTAATCC-3′). PCR reactions contained 2 × KAPA HiFi HotStart ReadyMix, 0.5 μM of each primer, and 10–12.5 ng genomic DNA. The amplification consisted of 95 °C for 3 min, followed by 25 cycles of 95 °C for 30 s, 55 °C for 30 s, and 72 °C for 30 s, with a final extension at 72 °C for 5 min. Amplicons were purified using AMPure XP beads (Beckman Coulter) and subjected to a limited-cycle PCR (8 cycles) to add dual Illumina indices and sequencing adapters, using the same temperatures and a final 5-min extension at 72 °C. Indexed products were purified again with AMPure XP, quantified, normalized, pooled, and sequenced on the Illumina MiSeq platform to generate paired-end reads. Raw sequence data were processed using QIIME 2 (18) Primers and adapters were trimmed with the cut adapt plugin, and sequence quality control, denoising, chimera removal, and amplicon sequence variant (ASV) inference were performed using the DADA2 plugin. Sequences containing ambiguous bases or <100 bp in length were removed. Taxonomic assignment was carried out using a Naïve Bayes classifier trained on the SILVA v138 reference database trimmed to the V3–V4 region and clustered at 99% identity, with a 70% confidence threshold (19). The resulting feature table was rarefied to 15,900 reads per sample prior to downstream analyses.

### 2.7 Data analyses

The experimental data were evaluated for homogeneity of variance using Levene’s test. When assumptions were met, parametric analyses (analysis of variance, ANOVA and the General Linear Model, GLM procedure of SAS) were performed. All data were analyzed in a completely randomized design. For growth performance and nutrient digestibility, the pen served as the experimental unit, whereas individual pigs were used as the experimental unit for blood profiles, and RR with treatments as fixed effects. Initial BW was included as a covariate for the analysis of final BW and hot carcass weight, whereas final BW was used as a covariate for dressing percentage. Hot carcass weight was included as a covariate in the analysis of backfat thickness. Statistical analyses were conducted using ANOVA and GLM procedure of SAS 9.4 M7 (SAS Institute Inc., Cary, NC, United States). Polynomial contrasts were applied to test for linear and quadratic effects of increasing dietary HEM levels (0, 0.05, and 0.10%). Statistical significance was declared at *p* < 0.05, and trends at 0.05 < *p* ≤ 0.10. The mathematical model employed in this study were as follows: Y*ij* = + *μ* + Ʈ*i* + *ϵ ij*. Herein, Y*ij*- stands for the dependent variables recorded in the *i*th treatment; μ, stand for population mean; Ʈ*i,* effect of *i*th treatment (1,2, and 3), and ϵ *ij*, stands for the *j*th observation on *i*th treatment.

Microbial diversity analyses were performed using QIIME2. Alpha diversity metrics (observed ASVs, Chao1, Shannon, Simpson, and Pielou’s evenness indices) were calculated using the “diversity” plugin, reflecting richness and evenness of microbial communities. Beta diversity was assessed using principal coordinate analysis (PCoA) based on unweighted UniFrac and Bray–Curtis distance matrices. Differentially abundant taxa among groups were identified using LEfSe, applying an LDA score threshold >2. Differences in microbial community structure were evaluated using the Mann–Whitney U test. Statistical significance was set at *p* < 0.05.

## 3 Results

### 3.1 Growth performance

The effect of dietary HEM supplementation on the growth performance of growing-finishing pig is shown in Table 3. During Phase 1 (i.e, from week 0–5), pig fed increasing HEM supplement had tended to improve (*p* < 0.1) G: F compared to CON, and there was no evidence of difference in their BW, ADG, and ADFI. However, during phase 2 (i.e, from week 5–10), and overall experimental period (i.e, from week 0–10), pigs fed increasing HEM supplement had linearly increased (*p* < 0.05) BW, ADG, ADFI, and tendency to improve (*p* < 0.1) G: F.

**Table 3:** Effect of dietary HEM supplementation on growth performance in grow-finishing pigs^1^.

Items	CON	TRT 1	TRT 2	SEM^2^	*P-*value^3^	
					Linear	Quadratic
Body weight, kg						
Initial	53.92	53.92	53.92	0.004	0.690	0.491
Week 5	81.28	82.21	82.78	0.77	0.179	0.844
Week 10	110.22^b^	112.70^ab^	115.35^a^	1.67	0.038	0.964
Initial—week 5						
ADG, g	781	808	825	22	0.176	0.838
ADFI, g	2,227	2,233	2,265	33	0.422	0.743
G: F ratio	2.865	2.773	2.757	0.039	0.059	0.426
Week 5—week 10						
ADG, g	827^b^	871^ab^	931^a^	26	0.008	0.800
ADFI, g	2525^b^	2597^ab^	2651^a^	48	0.041	0.782
G: F ratio	3.049	2.999	2.867	0.070	0.078	0.638
Overall						
ADG, g	804^b^	840^ab^	878^a^	24	0.038	0.962
ADFI, g	2,367	2,415	2,459	38	0.099	0.970
G: F ratio	2.959	2.889	2.815	0.051	0.054	0.977

^1^CON, Basal diet; TRT 1, Basal diet + 0.05% HEM; TRT 2, Basal diet + 0.10% HEM. ^2^Standard error of means. ^3a, b^Means in the same row with different superscripts differ significantly (*P* < 0.05).

### 3.2 Nutrient digestibility

The effect of dietary HEM supplementation on the nutrient digestibility of grow-finishing pig is shown in Table 4. At the end of week 10, pigs fed increasing HEM supplement had linearly increased (*p* < 0.05) N digestibility compared to CON. However, there were no differences observed on their DM and E digestibility.

**Table 4:** Effect of dietary HEM supplementation on nutrient digestibility in grow-finishing pigs^1^.

Items, %	CON	TRT 1	TRT 2	SEM^2^	*P-*value^3^	
					Linear	Quadratic
Week 10						
Dry matter	72.03	72.35	72.78	0.63	0.415	0.945
Nitrogen	69.33^b^	70.47^ab^	71.04^a^	0.39	0.006	0.555
Energy	70.87	71.02	71.15	0.49	0.685	0.980

^1^CON, Basal diet; TRT 1, Basal diet + 0.05% HEM; TRT 2, Basal diet + 0.10% HEM. ^2^Standard error of means. ^3a,b^Means in the same row with different superscripts differ significantly (*P* < 0.05).

### 3.3 Carcass characteristics

At the end of the trial, no significant differences were observed in any carcass characteristic parameters with increasing levels of HEM supplementation (Table 5).

**Table 5:** Effect of dietary HEM supplementation on carcass grade quality in grow-finishing pigs^1^.

Items	CON	TRT 1	TRT 2	SEM^2^	*P-*value	
					Linear	Quadratic
Carcass wg, kg	88.11	88.39	88.63	0.64	0.573	0.985
BFT, mm	20.10	20.17	20.41	0.62	0.718	0.909
1+, %	20.00	22.86	22.86	–	–	–
1, %	40.00	40.00	42.86	–	–	–
2, %	40.00	37.14	34.29	–	–	–

^1^CON, Basal diet; TRT 1, Basal diet + 0.05% HEM; TRT 2, Basal diet + 0.10% HEM. ^2^Standard error of means.

### 3.4 Biochemical and hematological parameters

The results of biochemical and hematological parameters of grow-finishing pigs supplemented with HEM are presented in Table 6. At the end of week 10, HEM-treated pigs showed linearly (*p* < 0.05) reduced cortisol levels. Also, linear reduction in HSP70 level and tend to reduce MDA than CON. However, biochemical variables such as glucose, insulin, SOD, and GPx levels were linearly increased (*p* < 0.05) in pigs fed increasing HEM supplementation with Nil effect on BUN, WBC and NE.

**Table 6:** Effect of dietary HEM supplementation on biochemical and hematological parameters in grow-finishing pigs^1^.

Items	CON	TRT 1	TRT 2	SEM^2^	*P-*value^3^	
					Linear	Quadratic
Week 10						
Cortisol, μg/dL	2.59^a^	2.20^ab^	2.04^b^	0.15	0.020	0.528
Glucose, mg/dL	75.17^b^	75.82^ab^	75.97^a^	0.33	0.016	0.947
BUN, mg/dL	12.4	11.7	11.2	0.60	0.165	0.891
Insulin, μU/mL	6.92^b^	7.20^ab^	7.69^a^	0.23	0.031	0.717
WBC, 10^3^/μL	19.36	19.08	18.91	0.58	0.595	0.940
Neutrophil, %	23.60	23.14	22.73	0.38	0.126	0.962
Lymphocyte, %	58.85	59.04	59.29	0.17	0.951	0.898
PCV, %	43.95^b^	44.34^ab^	45.01^a^	0.37	0.056	0.762
SOD, ng/mL	7.03^b^	8.45^ab^	9.61^a^	0.56	0.005	0.848
GPx, ng/mL	95.23^b^	98.36^ab^	99.10^a^	1.05	0.018	0.367
MDA, ng/mL	129.90^a^	128.18^ab^	127.14^b^	0.92	0.048	0.765
HSP70, ng/mL	10.94^a^	9.07^b^	8.95^b^	0.29	<0.0001	0.122

^1^CON, Basal diet; TRT 1, Basal diet + 0.05% HEM; TRT 2, Basal diet + 0.10% HEM. ^2^Standard error of means. ^3a,b^Means in the same row with different superscripts differ significantly (*P* < 0.05).

### 3.5 Respiratory rate and rectal heat

**Figure 2:**
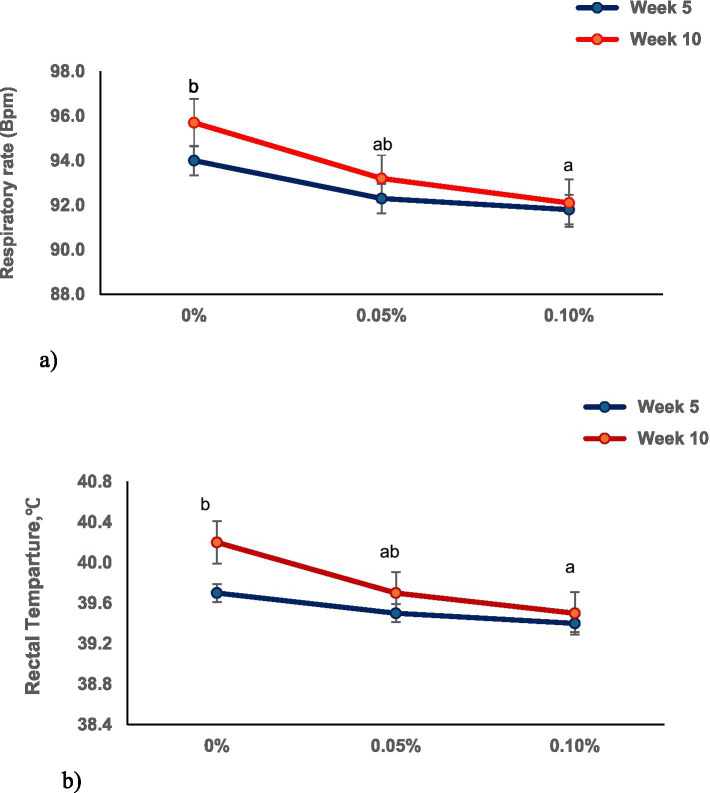
**(a,b)** Shows the effect of dietary HEM supplementation on the respiratory rate (RR) and rectal heat (RH) of grow-finishing pigs. ^1^CON, Basal diet; TRT1, Basal diet + 0.05% HEM; TRT 2, Basal diet + 0.10% HEM. At the end of week 5, HEM-treated pigs showed a linear reduction (*p* < 0.05) in RR and RH compared to CON. However, there was no evidence of difference (*p* > 0.10) observed in their RR and RH at the end of week 10 which proves that the animals were not under heat stress conditions during the study period.

At the end of week 5, HEM-treated pigs showed a linear reduction (*p* < 0.05) in RR and RH compared to CON (Figures 2a,b). However, there was no evidence of difference observed in their RR and RH at the end of week 10, which proves that the animals were not under heat stress conditions.

### 3.6 Fecal microbiome

**Figure 3:**
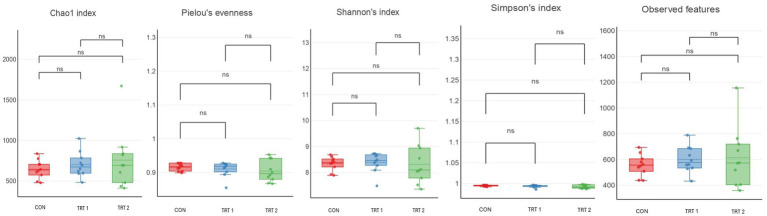
Illustrates the effect of HEM on alpha diversity in growing -finishing pigs. The boxplots represent the Chao1 index, Pielou’s evenness, Shannon’s index, Simpson’s index, and Observed features among three treatment groups. Each box indicates the interquartile range with the horizontal line representing the median, and whiskers showing variability outside the upper and lower quartiles. No significant differences (ns) were observed among groups for any of the diversity indices, indicating that dietary treatments did not significantly alter fecal microbial richness or diversity.

**Figure 4:**
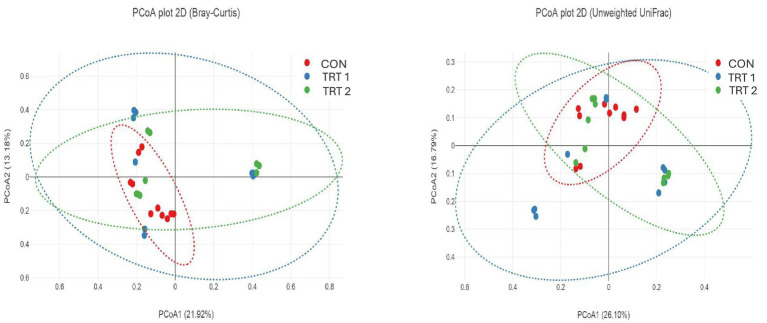
Illustrates the effect of HEM on beta diversity in growing- finishing pigs. The PCoA plots show microbial community clustering among three treatment groups based on Bray–Curtis dissimilarity and Unweighted UniFrac distance. Each point represents an individual sample, and the ellipses indicate 95% confidence intervals for each group. The axes (PCoA1 and PCoA2) represent the two principal coordinates explaining the highest variation in microbial community structure. The clustering patterns suggest no distinct separation among the groups, indicating that dietary treatments had no significant effect on the overall fecal microbial composition.

**Figure 5:**
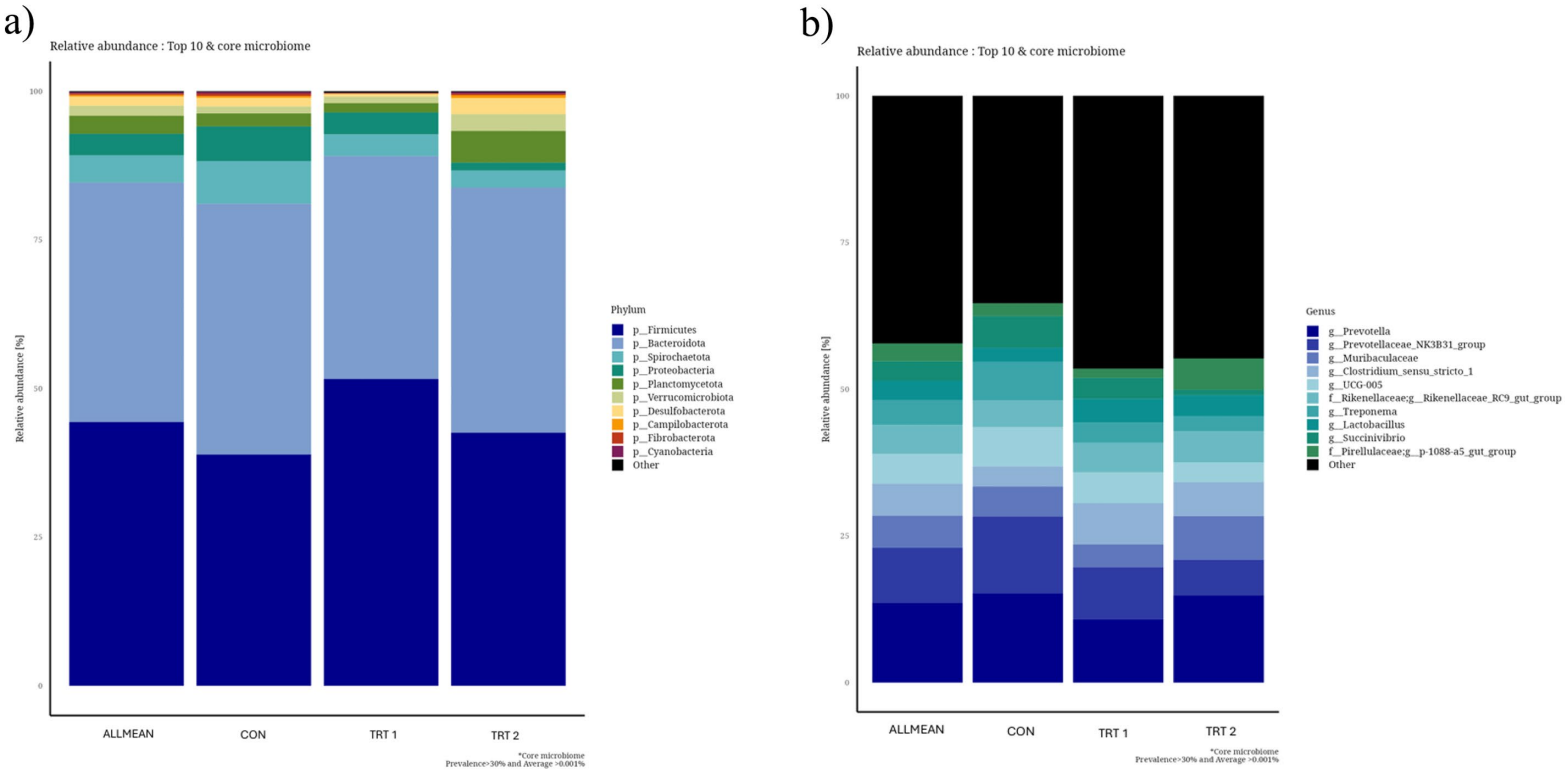
**(a)** Relative abundance of the top 10 bacterial phyla in the fecal microbiome of growing–finishing pigs. The stacked bar chart displays the proportional distribution (%) of dominant phyla across all samples. **(b)** Relative abundance of the top 10 bacterial genus in the fecal microbiome of growing–finishing pigs. The stacked bar chart displays the proportional distribution (%) of dominant phyla across all samples.

**Figure 6:**
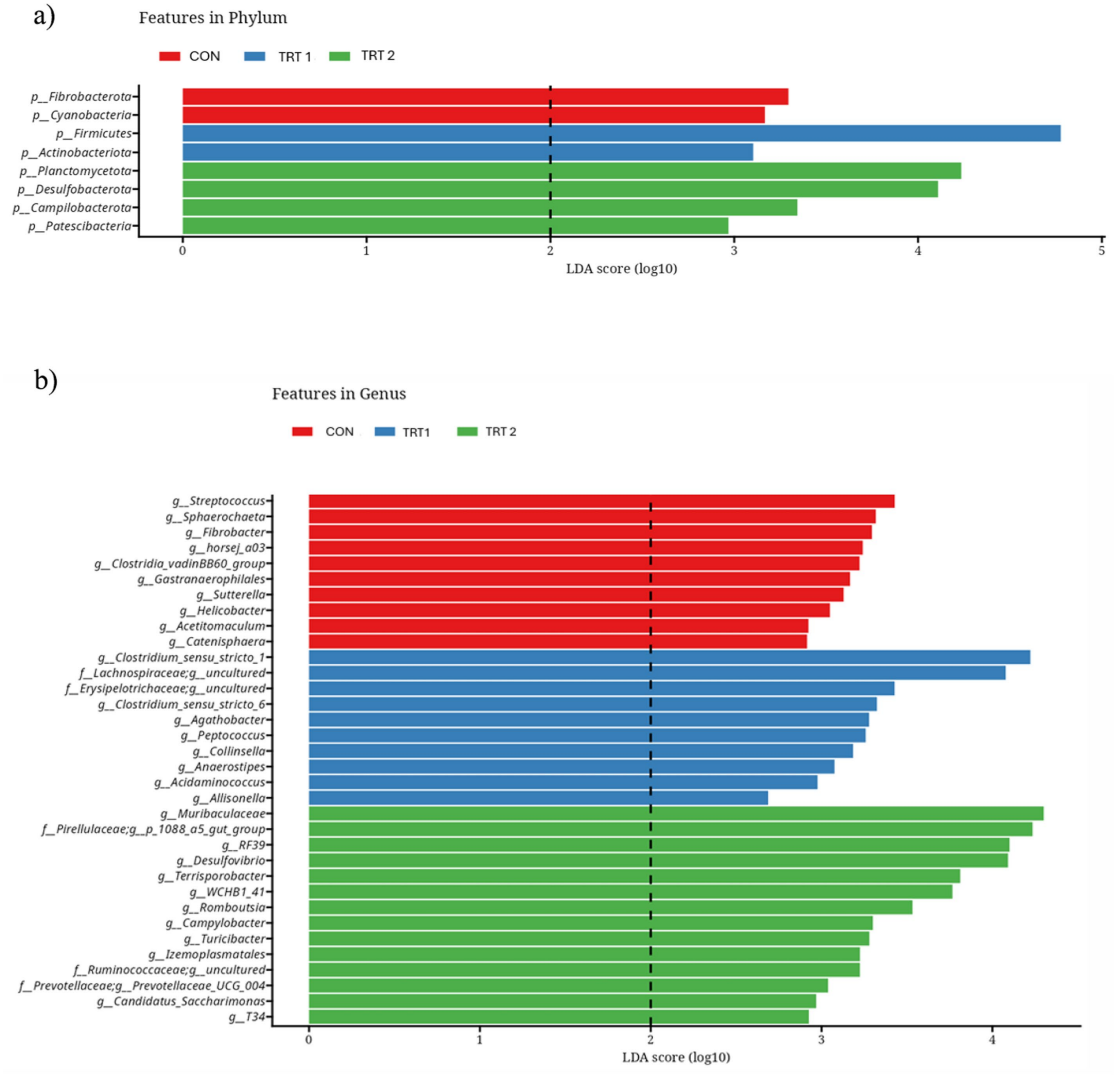
Linear discriminant analysis and effect size plot showing differentially abundant bacterial phyla **(a)** and genus **(b)** among treatment groups. Only taxa with LDA scores > 2.0 are presented, indicating microbial biomarkers significantly enriched in each treatment group.

Alpha diversity analyses, including Chao1 richness, Pielou’s evenness, Shannon’s index, Simpson’s index, and observed features, showed no significant differences among the treatment groups (Figure 3). Although TRT2 tended to show slightly higher values for Chao1, Shannon’s index, and observed features compared with the CON, these numerical increases did not reach statistical significance. Pielou’s evenness and Simpson’s index also remained comparable across all treatment groups. Beta diversity analyses using Bray–Curtis and unweighted UniFrac distances also revealed no clear separation among the treatment groups (Figure 4). In the Bray–Curtis PCoA plot, samples from all treatments clustered closely, indicating that the overall community composition remained largely similar across groups. A comparable pattern was observed in the unweighted UniFrac analysis, where the distribution of samples overlapped substantially, suggesting no significant differences in phylogenetic structure among treatments. Together, these findings demonstrate that dietary supplementation with herbal extracts did not markedly shift the overall microbial community structure in pigs. The relative abundance analysis of the top 10 phyla (Figure 5a) and core microbiome showed that the gut microbial community of pigs was dominated primarily by *Firmicutes* and *Bacteroidota* across all groups. In addition, minor phyla including *Spirochaetota, Proteobacteria, Planctomycetota, Verrucomicrobiota, Desulfobacterota, Campilobacterota, Fibrobacterota*, and *Cyanobacteria* were also identified. Notably, pigs in treatment groups exhibited a higher relative abundance of *Firmicutes* and *Bacteroidota*. At the genus level (Figure 5b) the gut microbiota of pigs was primarily dominated by *Prevotella,* followed by the *Prevotellaceae_NK3B31_*group, *Muribaculaceae, Clostridium_sensu_stricto_1,* and *UCG-005* and these genera were particularly lower in treatment groups. The minor genera including *Rikenellaceae_RC9_gut_group*, *Treponema*, *Lactobacillus*, *Succinivibrio*, and members of *Pirellulaceae* were also present at lower abundances. Furthermore, LEfSe analysis identified several phyla (Figure 6a) and genus (Figure 6b) that were differentially enriched among the treatment groups, with LDA scores greater than 2.0. In phyla, TRT1 group showed an enrichment of *Firmicutes* and *Actinobacteriota*, suggesting a moderate microbiome shift while, TRT 2 group pigs exhibited higher abundances of *Planctomycetota, Desulfobacterota, Campilobacterota*, and *Patescibacteria,* representing distinct microbial signatures associated with the higher level of HEM supplementation. In addition, TRT 1 showed significant enrichment in several genera, most notably the *uncultured Lachnospiraceae* feature, which displayed the highest LDA score across the entire analysis, along with *Erysipelotrichaceae _uncultured* and *Clostridium_sensu_stricto*_6.

## 4 Discussion

### 4.1 Growth performance and nutrient digestibility

Herbal feed additives have received increasing attention as alternatives to antibiotic growth promoters in swine nutrition due to their multifunctional bioactive properties, including antiviral, antibacterial, anti-inflammatory, and antioxidant activities (20). Beyond these effects, various herbal extracts have been shown to modulate the immune system, enhance digestive enzyme activity, and improve nutrient absorption, thereby supporting improved growth performance in pigs (21). Nevertheless, research evaluating the combined use of *S. baicalensis* and *L. japonica*as presented in the HEM additive remains limited, particularly in grow–finish pigs. In the present experiment, dietary supplementation with HEM linearly improved growth performance and tended to enhance G: F during Phase 2 and throughout the overall feeding period. These results suggest that pigs respond positively to increasing HEM levels, with performance benefits becoming more pronounced during the later stage of growth. Our findings are consistent with Liu et al. (22) who reported similar improvements in finishing pigs fed diets containing 0.025–0.05% herb extract. In agreement, Wang et al. (12), found improved performance in sows and their offspring with inclusion of *S. baicalensis* and *L. japonica* extract at 1.0 g/kg. Likewise, Zhao et al. (23) demonstrated improved weight gain, feed efficiency, and nutrient digestibility in weaned pigs supplemented with fermented medicinal plants containing *S. baicalensis*. Yan et al. (24) further supported the growth-promoting potential of complex herbal extract powders in growing pigs. However, inconsistent results have also been reported in the literature. For instance, Ao et al. (25) found no significant effects of herbal extract supplementation on growth performance in finishing pigs. Such inconsistencies may stem from differences in herbal composition, extraction processes, concentrations of active compounds, environmental conditions, or the physiological status of the animals. As highlighted by Wenk (26), herbal additives can influence feed palatability by enhancing taste and aroma, which in turn affects feeding behavior and digestive secretions in monogastric animals. Therefore, variability in palatability and bioactive profiles among herbal products may contribute to the diverse outcomes seen across studies. The increased N digestibility Our findings regarding nutrient digestibility are partially aligned with Collin et al. (27), who observed increased DM and N digestibility in pigs supplemented with herbal mixtures. One possible explanation for the increased feed intake observed in this study is the improved palatability resulting from HEM supplementation, while enhanced ADG and feed efficiency likely stem from the bioactive compounds present in *S. baicalensis* and *L. japonica* as have been associated with improved digestive function, reduced inflammation, and enhanced nutrient utilization, which collectively support better growth and overall physiological status.

### 4.2 Respiratory rate and rectal temperature

Herbal extracts rich in phytochemicals have gained considerable attention as feed supplements for alleviating or mitigating stress in pigs (28). Under HS, when ambient temperatures exceed the upper critical limit of the thermoneutral zone, pigs initiate several physiological responses aimed at reducing internal heat production and maintaining homeostasis (29). These adaptive mechanisms include the redistribution of blood flow toward the skin surface (27), increases in rectal temperature (30), and elevated RR, all of which facilitate evaporative heat loss and reduce heat retention (29, 31). Previous research has shown that pigs exposed to HS frequently reduce feed intake as a strategy to lower metabolic heat production (32, 33). Heat acclimation is a progressive and dynamic process, and its effectiveness directly influences physiological functions, tissue synthesis, and tissue deposition (34, 35). As pigs lack functional sweat glands (36), increases in RR serve as a primary mechanism for dissipating excess body heat through respiratory evaporation (37). Consequently, RR and rectal temperature are widely recognized as sensitive physiological indicators of HS in pigs, particularly when environmental temperatures exceed 25 °C (38). Although these homeostatic responses help the animal cope with thermal challenges, they often lead to compromised productivity, with feed intake being the most consistently affected parameter (39). In the present study, pigs raised under hot and dry environmental conditions exhibited a linear reduction in RR and RH at the end of week 5 in response to increasing levels of HEM supplementation, indicating improved physiological stability during the early stage of the trial. However, by week 10, no differences in RR or RH were observed between treatments, suggesting that pigs had successfully acclimated to the prevailing thermal environment regardless of diet. This pattern aligns with previous reports by Cervantes et al. (40) and Renaudeau et al. (32), who documented similar reductions in RR and rectal temperature as pigs gradually acclimated to HS conditions. The early reduction in RR observed in HEM-supplemented pigs may be attributed to the anti-inflammatory, antibacterial, antiviral, and anti-stress properties of *S. baicalensis* and *L. japonica*, which can mitigate respiratory tract irritation and support thermoregulatory efficiency (41).

### 4.3 Fecal microbiome

The gut microbiota plays an essential role in maintaining intestinal homeostasis and supporting overall host health (42). Substantial evidence has demonstrated that higher microbial diversity contributes to improved immune regulation and resilience against disease (43). However, previous studies have also shown that the effects of herbal supplementation on gut microbial diversity may vary depending on the physiological stage of the animal. For instance, Xu et al. (44) observed that Chinese herbal mixtures improved alpha diversity only during early post-weaning, whereas Li et al. (45) noted that microbial diversity indices naturally fluctuate with age. These findings suggest that the influence of herbal additives on microbial richness and evenness may be subtle and may depend on both the dosage and the developmental stage of pigs. Although HEM supplementation did not markedly alter overall microbial diversity in the present study, the observed taxonomic shifts at the phylum and genus levels are biologically relevant. Firmicutes and Bacteroidota remained the dominant phyla across treatments, consistent with the typical gut microbiota profile of healthy pigs reported by Isaacson and Kim (46). The higher relative abundance of Firmicutes observed in HEM-supplemented groups is noteworthy because Firmicutes have been associated with enhanced energy harvest, improved nutrient processing, and increased fat deposition in animals (47, 48). This suggests that the modulation of these bacterial populations by HEM may contribute to improved growth performance, possibly by promoting more efficient metabolism and nutrient utilization. To further clarify the relationships between microbial composition and performance traits, partial correlation analysis was employed to account for potential confounding factors (49). The positive association between ADG and Firmicutes abundance supports previous findings in ruminants, where Firmicutes were linked to improved daily gain (50). This relationship reinforces the idea that even modest shifts in microbiota composition without major changes in diversity can have meaningful physiological implications for host metabolism. Additionally, the increase in beneficial genera such as *Lactobacillus* is known to exert antioxidant activity, reduce oxidative stress, and support epithelial health by lowering ROS (51).

### 4.4 Biochemical and hematological parameters

Blood biochemical and hematological indices serve as important indicators of physiological stability and health status in animals (52). Among these, cortisol is widely used as a primary biomarker of stress. However, Gross and Siegel (53) suggested that neutrophil-to-lymphocyte ratios may provide more stable assessments of chronic stress responses. The reduced cortisol concentrations observed in pigs receiving HEM supplementation, together with stable leukocyte profiles, indicate that the herbal mixture may attenuate stress responses without triggering immune disruption. Such effects may be attributed to the regulatory influence of bioactive phytochemicals on the hypothalamic–pituitary–adrenal (HPA) axis, which governs the release of stress-related hormones. Because herbal extracts are known to modulate HPA activity and prevent neuroendocrine imbalance, it is plausible that HEM helped maintain a more controlled stress response in pigs exposed to hot environmental conditions. Oxidative stress is another critical factor affecting metabolic function and productivity, particularly during heat exposure. Lipid peroxidation, often assessed via MDA concentration, increases when oxidative damage is elevated (54, 55). The lower MDA levels in HEM-supplemented pigs suggest that the antioxidant constituents of *S. baicalensis* and *L. japonica* may have enhanced endogenous antioxidant capacity, thereby limiting cellular oxidative damage during summer heat. This interpretation contrasts with the findings of Montilla et al. (56), who reported sharp increases in MDA following acute heat stress, highlighting the potential protective role of sustained phytochemical supplementation in mitigating chronic oxidative challenges. The concurrent increase in antioxidant enzyme activities further supports the likelihood that HEM contributed to strengthening enzymatic defenses against ROS. Heat stress also commonly affects hematological indices, with reductions in hemoglobin and hematocrit frequently linked to erythrocyte fragility and reduced erythropoiesis caused by elevated respiratory activity (51). Studies evaluating herbal or phytochemical-rich feed ingredients have reported improvements in hematological stability under stressful conditions (57), suggesting that plant-derived antioxidants may help preserve red blood cell integrity. Heat shock proteins (HSPs) play a critical role in maintaining cellular homeostasis by facilitating protein assembly and disassembly, promoting correct protein folding and unfolding, and refolding damaged proteins (58). Under stress conditions, HSPs have been shown to inhibit abnormal protein aggregation, assist in the intracellular transport of misfolded proteins for degradation, and support tissue repair mechanisms (59). In this study, HEM treated pigs linearly reduced heat shock protein compared to CON which did not agree with Parkunan et al. (60) who noted significantly increased HSP70 expression in Large White Yorkshire breeds. This discrepancy is likely attributable to breed differences and environmental adaptation, as earlier studies have shown that early or mild heat exposures may not consistently elevate HSP expression (61). The reduction in HSP70 with HEM supplementation suggests that the anti-inflammatory and antioxidant constituents of the herbal extract blend may help alleviate the cellular burden imposed by heat, thereby reducing the need for stress-induced chaperone activity.

## 5 Conclusion

Overall, the findings of this study indicate that dietary supplementation with 0.05 to 0.10% of the herbal extract mixture (HEM) can significantly improve growth performance, enhance nutrient digestibility, alleviate oxidative stress, and mitigate the adverse effects of heat stress in pigs. These outcomes suggest that HEM supplementation represents a promising nutritional strategy to support swine production under hot and dry climatic conditions. Furthermore, the results support the hypothesis that HEM exerts a protective effect by modulating stress responses, strengthening antioxidant defense mechanisms, and preserving metabolic homeostasis, thereby contributing to improved health and welfare in pigs.

## Data Availability

The original contributions presented in the study are publicly available. This data can be found here: NCBI repository, accession number PRJNA1441127, https://www.ncbi.nlm.nih.gov/bioproject/PRJNA1441127.
